# Factors Affecting Digital Tool Use in Client Interaction According to Mental Health Professionals: Interview Study

**DOI:** 10.2196/44681

**Published:** 2023-07-10

**Authors:** Lauri Lukka, Veli-Matti Karhulahti, J Matias Palva

**Affiliations:** 1 Department of Neuroscience and Biomedical Engineering Aalto University Espoo Finland; 2 Faculty of Humanities and Social Sciences University of Jyväskylä Jyväskylä Finland; 3 Neuroscience Center Helsinki Institute of Life Science University of Helsinki Helsinki Finland; 4 Centre for Cognitive Neuroimaging Institute of Neuroscience and Psychology University of Glasgow Glasgow United Kingdom

**Keywords:** clinical practice, digital mental health interventions, intervention design, mental health applications, mental health professionals, teletherapy, mobile phone

## Abstract

**Background:**

Digital tools and interventions are being increasingly developed in response to the growing mental health crisis, and mental health professionals (MHPs) considerably influence their adoption in client practice. However, how MHPs use digital tools in client interaction is yet to be sufficiently understood, which poses challenges to their design, development, and implementation.

**Objective:**

This study aimed to create a contextual understanding of how MHPs use different digital tools in clinical client practice and what characterizes the use across tools.

**Methods:**

A total of 19 Finnish MHPs participated in semistructured interviews, and the data were transcribed, coded, and inductively analyzed.

**Results:**

We found that MHP digital tool use was characterized by 3 distinct functions: communication, diagnosis and evaluation, and facilitating therapeutic change. The functions were addressed using analog tools, digitized tools that mimic their analog counterparts, and digital tools that use the possibilities native to digital. The MHP-client communication included various media alongside face-to-face meetings, the MHPs increasingly used digitized tools in client evaluation, and the MHPs actively used digitized materials to facilitate therapeutic change. MHP tool use was generally characterized by adaptability—it was negotiated in client interactions. However, there was considerable variance in the breadth of MHPs’ digital toolbox. The existing clinical practices emphasized MHP-client interaction and invited incremental rather than radical developments, which challenged the achievement of the scalability benefits expected from digital tools.

**Conclusions:**

MHPs use digitized and digital tools in client practice. Our results contribute to the user-centered research, development, and implementation of new digital solutions in mental health care by classifying them according to their function and medium and describing how MHPs use and do not use them.

## Introduction

### Background

Mental disorders are the leading cause of disease burden worldwide [1]. However, a substantial number of people with mental disorders fail to receive adequate support and treatment for their challenges [2]. It is believed that digital technologies can increase the effectiveness, accessibility, and cost-effectiveness of existing treatments, which is a considerable motivation for their development [3-7].

Mental health professionals (MHPs) play a considerable role in how mental disorders are treated and which digital tools and materials are used in clinical practice. They act as gatekeepers for web-based therapies [8] and exercise their influence by recommending digital materials, platforms, and treatments to their clients [9]. Thus, alongside their clients, MHPs constitute a second key user group [10] whose attitudes and needs are vital to understand when designing and developing new digital tools.

MHP and client needs relate through the so-called therapeutic alliance. Bordin [11] describes that it consists of three factors: (1) a positive attachment bond between the MHP and their client, (2) their shared agreement on the therapy goals, and (3) pursuing these goals through tasks in therapeutic interaction. The therapeutic relationship has been found to substantially contribute to the effectiveness of therapy [12,13]. Today, digital tools present changes and possibilities for the therapeutic alliance [14-16]—telehealth solutions facilitate the contact between the MHP and their client, and in counseling, the MHP may facilitate the change through complementary digitized materials, digital therapies, and mobile apps. The therapeutic alliance is becoming digitally enhanced.

### MHP Digital Tool Use in Client Practice

#### Telehealth Solutions

The COVID-19 crisis has substantially changed how MHPs interact with their clients. Before the crisis, only a minority of the interactions occurred remotely. A 2018 published survey found that 57% of US psychologists did not engage in telecounseling, with only 6% delivering >6 hours of telecounseling per week [17]. Landlines and mobile phones were considerably more commonly used. In Portugal, Mendes-Santos et al [9] had similar findings: 30% of psychologists used digital technology to support their clients, most often via telephone, email, and SMS text messages; only 9% used videoconferencing. Such low use likely reflects the numerous barriers to using telehealth solutions, including the perceived dehumanization of the therapeutic environment, client and clinician suitability factors, and the prohibitive costs of the solutions, as well as issues with reimbursement, confidentiality, and data protection [18,19]. However, the pandemic has forced therapists to offer their services remotely [20-22], and today, digital media increasingly facilitate client contact.

#### Digital Materials

Clients can use psychoeducational materials independently to alleviate their psychiatric symptoms, such as depression [23]. MHPs also often recommend complementary web-based materials to be accessed between therapy sessions [9,24], including websites, forums, blogs, social media, and support groups. The need for recommendations exists as not all abundant web-based materials comply with and reflect the best treatment practices [3]. To help both MHPs and their clients navigate web-based materials, digital mental health hubs have been created. Canadian eMentalHealth.ca, for example, provides information on mental health, self-assessment forms, and contact points to health care, and the 2 million annual users generally find the service positive [25]. In Finland, MentalHub (in Finnish, “Mielenterveystalo”), developed by the Helsinki University Hospital (HUS), serves a similar purpose. It provides psychoeducation, self-guided treatments, symptom navigators, service directories, and internet therapies [26].

#### Digital Mental Health Interventions

Comprehensive and structured digital treatment programs have also been developed. These emerging therapies have been called computer-assisted therapy [27] and internet-delivered psychological treatments [28]; we use the concept of digital mental health interventions (DMHIs) [29]. A recent meta-analysis found that therapist-supported internet-based interventions yield similar effects as face-to-face cognitive behavioral therapy (CBT) [30]. Indeed, MHP contact is beneficial in motivating and engaging the client in the digital intervention. DMHIs with therapist support are more effective than without it [31,32]. Similar to traditional face-to-face therapy, the quality of the mediated therapeutic alliance during the DMHI contributes to treatment outcomes [33]. Moreover, it appears that the richness of the contact facilitates treatment results—face-to-face support is more effective than telephone support, which is more effective than email support [27].

Unguided, guided, and blended therapies have been actively developed and used. An example of a guided DMHI is the HUS-provided, physician-referred 12-session CBT program for generalized anxiety disorder [34]. The program is theoretically based on several models of anxiety, trained therapists offer support through the program using asynchronous messages, and the program includes persuasive elements such as simulation and reminders to increase client engagement. HUS has developed internet therapies for other mental disorders as well, such as depression, attention-deficit/hyperactivity disorder, and bipolar disorder [35], which are referred to in this research as “national DMHI.” Blended therapies combine both digital and face-to-face interaction [36]. In the United Kingdom, Stawarz et al [37] reported a blended approach comprising initial face-to-face meetings, subsequent web-based therapy sessions, and independent work by the client between sessions. Generally, MHPs are more favorable toward blended approaches than unguided therapies [8,38-40]. This reflects their profession that emphasizes the importance of the healing therapeutic relationship which frames discussions on digital tools.

#### Mental Health Apps

Finally, the rapid proliferation of smartphones has brought health care to the clients’ pockets, and the use of mental health apps is growing [41]. In contrast to the DMHIs that are often developed and delivered in association with health care organizations and may also be clinician-prescribed and reimbursed, mental health apps are often distributed directly to consumers and may be used independently of health care contact [42]. In some countries, 50% of mental health service–using youth [43] and 10% of outpatient psychiatry clinic patients have used mental health apps [44]. An example of a commercial mental health app is “Calm,” which offers a mindfulness meditation intervention that can reduce stress [45] and increase well-being [46]. An example of a Finnish publicly funded mental health app is “Chillaa,” which is targeted to youth aged 13 to 15 years and aims to reduce stress and social anxiety [47].

Mental health apps may complement therapies [48], and some MHPs recommend them to their clients. In Portugal, 28% of psychologists had recommended apps to their clients [9], whereas in examining mental services for the youth, Bell et al [43] found that 84% of clinicians had recommended apps to their clients. However, there have been considerable concerns regarding the quality and evidence base of the app content [49-53] as well as their privacy [54], which, together with the lack of guidance [55], diminish their credibility and slow their adoption in mental health care [56].

In summary, MHPs use various digital tools in their client practice. Moreover, the use of fully digital and blended DMHIs is growing—a trend that is occurring alongside the proliferation of mobile mental health apps. The growing adoption of digital technologies in society and health care and the specific changes in the digital mental health landscape frame MHP attitudes regarding digital tools.

### Study Aims

Previous research has examined MHP adoption of digital technologies, their attitudes toward them, and the factors influencing their implementation. However, less attention has been paid to *how* MHPs use digital tools in client practice. We posit that a qualitative, user-centered approach can provide rich, in-depth insights into the MHP working context and their attitudes, needs, preferences, and behavior regarding digital tools [57-60]—factors vital to their design, development, and implementation.

We followed the hypothesis-generating qualitative research tradition [61] and focused on how MHPs describe their client practice [62]. How we conceptualized qualitative analysis in this study reflected a perspective aptly described by Fossey et al [63]:

Qualitative research aims to address questions concerned with developing an understanding of the meaning and experience dimensions of humans’ lives and social worlds. Central to good qualitative research is whether the research participants’ subjective meanings, actions, and social contexts, as understood by them, are illuminated.

Initially, the study was planned to better understand MHP views and needs regarding DMHIs with game elements. The aim was broken down into 2 areas the MHPs were familiar with: how they perceived and used digital tools and how they viewed digital games in client practice. In the very first interviews, it was discovered that these 2 areas were separate—digital tool use was MHP initiated, whereas playing digital games addressed client behavior. Thus, this study focused on the first area, which was further broken down into two specific research questions (RQs): How do MHPs use different digital tools in client practice? (RQ 1) and What characterizes MHPs’ digital tool use in client practice in general? (RQ 2).

To further the study aims, 19 semistructured interviews with Finnish MHPs were conducted, analyzed inductively, and reported under the 2 RQs.

## Methods

The study was conducted in 3 phases: recruitment, interview, and analysis. A survey was used to gather background information, whereafter MHPs were invited to a semistructured interview, and the transcribed interview data were analyzed inductively.

### Ethics Approval

The study was approved by the Aalto University research ethics committee (D/508/03.04/2022), and the research design was preregistered in the Open Science Framework [64].

### Data and Sampling

The guiding principle in participant recruitment was maximum local variation—the recruitment aimed to gather a diverse sample of Finnish MHPs with various educational backgrounds, who worked in different organizational contexts, and with various client populations in health care. Finnish health care is primarily public and organized by municipalities [65] and tiered into low-threshold basic-level services for those with less severe disorders and specialized psychiatric services for clients with more severe disorders. The Finnish Student Health Service provides mental health care services for university students [66], and occupational health care provides health care services and brief counseling for the workforce. Rehabilitative psychotherapy delivered by licensed psychotherapists can be reimbursed for up to 3 years [67].

The study participants were recruited through social media, local professional association channels, and health care organizations. Snowballing was used to recruit professionals from the expert networks of the interviewees. The participant recruitment advertisements highlighted that the interviewees were not expected to have experience using digital tools and therapies to welcome participants with various levels of experience. The study inclusion criteria were (1) being a licensed health care professional, (2) working with mental health, and (3) having at least one customer weekly. Interviews were conducted with a Finnish interview frame; thus, non–Finnish-speaking participants were not included. The recruitment and interviews took place between May 11, 2022, and September 8, 2022.

A recruitment link shared on the web led the possible participant to a web-based survey in Finnish created using Webropol software (Webropol Limited) and included an informed consent form and privacy notice. According to the service use statistics, 833 people opened the digital questionnaire, of whom 109 (13.1%) began to answer it, with 80 (73.4%) of the 109 MHPs completing it. A total of 34 respondents indicated their willingness to participate in the interviews, of whom 24 (71%) were contacted by the first author via email. In total, 9% (3/34) of the respondents did not respond to the inquiry, and 6% (2/34) withdrew before the interview: one because of a lack of time and another because of their considerable prejudices against the topic.

The concept of saturation [68] was used to evaluate the sufficiency of the sample. The first author evaluated saturation using analytic memoing conducted after each interview [69] and during coding, which was carried out in parallel to the interviews. After approximately 12 interviews, the first author found that they contributed less and less new information. After a collaborative reflection of the analytic memos and initial codes with the second author, it was deemed that the data from 19 interviews were sufficient to answer the RQs. Therefore, not all 34 respondents willing to be interviewed were contacted. The sample size of 19 is aligned with a recent systematic review that found that 9 to 17 interviews reached saturation in homogenous study populations such as ours [70], as well as previous research on interview sample sizes [71]. The characteristics of the interviewees are described in Table 1 and individually in Table 2.

**Table 1:** Characteristics of the interviewees (n=19).

Variable and category		Values
**Gender, n (%)**		
	Woman	13 (68)
	Man	6 (32)
**Age range (years), n (%)**		
	18-29	1 (5)
	30-39	3 (16)
	40-49	6 (32)
	50-59	7 (37)
	60-69	2 (11)
**Working status, n (%)**		
	Full time	15 (79)
	Part time	4 (21)
	Not working	0 (0)
Years of mental health work experience, mean (SD; range)		18 (13.7; 1-43)
Hours of customer work per week, mean (SD; range)		18 (5.9; 9-30)
**Education (multiple options may be chosen), n (%)**		
	Practical nurse	2 (11)
	Nurse	7 (37)
	Psychologist	11 (58)
	Psychotherapist	7 (37)
	Social worker	1 (5)
	Other	6 (32)
**Context of client work, n (%)**		
	Specialized health care	12 (63)
	Student health care	1 (5)
	Independent practice	6 (32)
**Clients with..., n (%)**		
	Mild mental disorders	1 (5)
	Moderate mental disorders	7 (37)
	Severe mental disorders	11 (58)

**Table 2:** Individual characteristics of the interviewees (n=19).

Number	Clinical education	Working context	Role	Clients	Digital tools used and discussed in the interview
1	Occupational therapist	Specialized health care	Psychosocial treatment	Adults with mood disorders	TT^a^ and MA^b^
2	Psychologist psychotherapist	Independent practice	Psychotherapy	Adults with mood disorders	TM^c^ and TT
3	Psychologist psychotherapist	Specialized health care	Psychological evaluation	Adults with neuropsychological challenges	TM, TT, WM^d^, and digital psychological tests
4	Nurse	Specialized health care	Psychosocial treatment	Adults with psychosis or prodromal symptoms	TM, TT, WM, and digital cognitive rehabilitation therapy
5	Nurse psychotherapist	Specialized health care	Evaluation and consultation	Youth with psychological symptoms	TM, TT, WM, and MA
6	Psychologist psychotherapist	Student health care	Psychological treatment	Students with sexuality-related challenges	TT, WM, and digitized questionnaires
7	Nurse	Specialized health care	Evaluation and psychosocial treatment	Adults with mood disorders	TM, TT, and WM
8	Psychologist psychotherapist	Independent practice	Psychotherapy	Adults with mood disorders	TT and client-introduced apps
9	Nurse	Specialized health care	Evaluation and psychosocial treatment	Adults with neuropsychological challenges	TM and TT
10	Psychologist	Specialized health care	Psychological evaluation and psychosocial treatment	Older adults with psychiatric challenges	TM, TT, and WM
11	Nurse	Specialized health care	Care coordination and psychosocial treatment	Older adults with psychiatric challenges	TM, TT, WM, and digitized questionnaires
12	Nurse	Psychiatric inpatient ward	Care coordination and psychosocial treatment	Adults with psychiatric disorders	TT and WM
13	Nurse	Specialized health care	Evaluation and psychosocial rehabilitation	Adults with psychotic disorders	TM, TT, and WM
14	Psychologist	Specialized health care	Psychosocial treatment and psychological evaluation	Adults with lowered ability to work	TM and WM
15	Psychologist	Specialized health care	Psychosocial treatment	Adults with mood disorders	TM, TT, WM, and acted as a DMHI^e^ therapist
16	Psychologist psychotherapist	Independent practice	Psychotherapy	Adults with psychiatric disorders	TM and TT
17	Psychologist psychotherapist	Independent practice	Psychotherapy	Adults with psychiatric disorders	TT and client-introduced apps
18	Psychologist	Independent practice	Neuropsychological rehabilitation	People with neuropsychological problems	TT, WM, and rehabilitation software
19	Psychologist psychotherapist	Psychotherapy center	Psychotherapy	People with psychological trauma-related problems	TT and therapy centers’ digital materials

^a^TT: teletherapy.

^b^MA: mobile app.

^c^TM: telephone or messaging.

^d^WM: web-based materials.

^e^DMHI: digital mental health intervention.

### Semistructured Interview

The interview was semistructured [72], focusing on the MHPs’ subjective experiences with digital tools in their professional context in client interaction. Although keeping with the RQs, attention was paid to ensuring that the interviews retained their flexibility and accommodated the variance in the interview contexts. Using the typology by McIntosh and Morse [73], the interview was primarily descriptive and interpretive, focusing on discovering the interviewees’ experiential world as opposed to testing a particular theory aligned with consequent inductive data analysis. The interview guide is presented in Multimedia Appendix 1.

The first author conducted the interviews remotely using Zoom (Zoom Video Communications). He is a clinical psychologist and service designer experienced in conducting interviews and versed in clinical mental health care. The interviews were recorded after verbally confirming the interviewee’s consent (according to national research guidelines) and transcribed verbatim for analysis. The interview durations ranged from 47 to 83 minutes, with an average duration of 56 (SD 9) minutes. The total interview data duration was 18 hours 9 minutes, which led to transcribed materials of 96,707 words.

### Inductive Data Analysis

The data analysis was conducted in 2 parts reflecting the hierarchical nature of the RQs: the use of specific digital tools (RQ 1) is subordinate to digital tool use in general (RQ 2). Through this approach, we pursued transparency to the often nebulous theme generation and to establish rigor in the research [74] by showing the relationship between specific digital tool use and higher-order themes. In this study, we defined themes as patterns in the data and followed the definition by DeSantis and Ugarriza [75]: “A theme is an abstract entity that brings meaning and identity to a recurrent experience and its variant manifestations. As such, a theme captures and unifies the nature or basis of the experience into a meaningful whole.” Thus, the theme exhibited both unity across participants and internal variance.

The interview data were analyzed inductively to establish the themes bottom-up instead of deductively testing a particular theory. This aimed to ensure that the interviewees’ perspectives came across in the analysis rather than those of the researchers. We are aware that some components of this analysis approach—such as assessing saturation or following COREQ (Consolidated Criteria for Reporting Qualitative Research) guidelines (refer to later sections)—are not promoted in thematic analysis. Otherwise, the data analysis closely followed the 6-step process by Braun and Clarke [76] described as thematic analysis. In the first step, “Familiarizing yourself with the data,” the first author transcribed the data verbatim and then confirmed the transcription accuracy by relistening to the interview tapes with the written transcription, which further familiarized him with the data. In the second step, “Generating the initial codes,” the first author coded all the data using ATLAS.ti software (version 22; ATLAS.ti GmbH), allowing for the initial organization of the data into categories. Then, the first author conducted the third, fourth, fifth, and sixth steps of the analysis—“Searching for themes,” “Reviewing themes,” “Defining and naming themes,” and “Producing the report”—per the 2 RQs. Reflexivity was ensured by the ongoing reflection of code and theme generation through the first author’s clinical background and position and the full context of all the data.

To answer RQ 1, the first author started to search for meanings by categorizing all instances where interviewees discussed specific digital tool use, adding up to 349 codes. For example, all the instances in which the interviewees reflected on the different ways in which they used telephone, SMS text messages, WhatsApp, Skype, and Zoom to stay in touch with their clients were categorized per medium. These categories were further grouped into a higher-order category of “Communication.” In total, 2 other categories were established: tool use related to psychiatric evaluation and diagnostics and tool use to facilitate therapeutic change. Because of the descriptive, pragmatic nature of RQ 1, we chose to report these 3 categories as domain summaries—“summaries of the range of meaning in the data related to a particular topic or ‘domain’ of discussion” [77]. The domains comprised the 3 functions the digital tools served, which were identified from the data. The 3 categories were reviewed to ensure that they included all the digital tools discussed in the interview, and they were named and reported in the *Results* section.

After analyzing digital tool use for RQ 1, the first author began the development of themes for RQ 2. A total of 335 initial codes included interviewees’ reflections on how they viewed and used digital tools in their client practice in general, and these initial codes were searched for themes. For instance, the recurring notion that digital tools do not replace face-to-face connections was reflected on, similarly to mentions of how digital tools may alleviate resource problems in psychiatry and how it is essential to consider the client’s needs. This search led to the establishment of 3 themes. MHP flexibility in client interaction recurred in almost all interviews, and it was also the most frequent coding category. Thus, the first theme described the client-centered clinical approach that unified the participants. The second theme contrasted with the first by highlighting the variance in the MHP digital toolbox. Finally, a third theme was established by examining how digital tools influenced MHP work. The names of the themes were refined several times to ensure that they captured the essence of the interviewees’ accounts. Particular attention was paid to ensuring that the themes had internal consistency and described the whole data set.

Further efforts were made to ensure that the data analysis was reliable. The first and second authors met 2 times to reflect on the data analysis, review the themes, and name them. The first author translated the interviewees’ quotes from Finnish into English, and another researcher (Maria Vesterinen) reviewed the translations, which led to minor clarifications. The results were annotated with interview and paragraph references (eg, #1:100) to facilitate transparency. Member checking [78] was used to ensure that the interpretations made in the study represented the notions of the MHPs. The draft version of the manuscript was sent to 5 MHPs in October 2022 and November 2022: a total of 2 (40%) MHPs who were interviewed, 2 (40%) MHPs who were employed at HUS, and 1 (20%) independent MHP. Their feedback supported the findings, and only minor clarifications were made based on it. Finally, the researchers confirmed that describing and analyzing the study results conformed to the COREQ guidelines [79].

## Results

### How Do MHPs Use Different Digital Tools in Client Practice? (RQ 1)

#### Overview

We found that MHPs used digital tools in client practice for three functions: (1) diagnosis and evaluation and (2) counseling, both of which necessitate (3) communication with the client (Table 3). This evaluation aimed to create an understanding of the client’s challenges and disorders to guide treatment. It typically consisted of interviews and questionnaires complemented with psychological testing when a more thorough understanding of the client’s problems and cognition was required. Counseling sought to alleviate the clients’ symptoms and helped them cope with their challenges. Depending on the MHP’s education and role, it may be psychosocial support in a clinic or psychiatric ward, neuropsychological rehabilitation, short-term therapy, or psychotherapy. In this paper, the term *counseling* is used to refer to all modes of psychosocial support and treatment. Both evaluation and counseling require contact with the client—communication.

**Table 3:** The 3 distinct functions that characterize mental health professional digital tool use in client interaction. The functions can be served through analog, digitized, or digital solutions, of which examples are provided.

	Medium		
Function	Analog	Digitized	Digital
Communication	Face-to-face interaction and written letters	Telephone, emails, messaging, and teletherapy with audio and video connection	Teletherapy with advanced features such as virtual reality and avatars
Diagnosis and evaluation	Pen-and-paper questionnaires and psychological tests	Sending questionnaires via email and filling questionnaires on the web	Responsive and gamified tests and integration of various data sources
Creating therapeutic change	Brochures, printed materials, and handouts	Sharing materials via email or through web-based information portals	Interactive DMHIs^a^, mobile apps, and serious games

^a^DMHI: digital mental health intervention.

We argue that MHP functions reflect the nature of their profession and its practices. Thus, the need for communication with the client, creating an understanding of their challenges through evaluation, and supporting them is likely to remain constant over time, whereas *how* MHPs achieve these functions may evolve and change. The change is driven by technological advancement, which we describe on a continuum from analog to digitized and digital media. By analog, we refer to nondigital media; by digitized media, we refer to an analog medium converted into digital without substantial changes or additions. In contrast, digital refers to media that use the possibilities native to digital. We acknowledge that the lines between the 3 are not always fully clear and keep evolving; however, the more detailed ontological discussion must be left elsewhere.

The 3 MHP functions may be implemented in analog, digitized, or digital media. Regarding communication, the telephone digitizes verbal interaction, and emails and SMS text messages digitize written communication. In contrast, digital teletherapy solutions can change the nature of the interaction by, for instance, augmenting the conversation with interactive materials and features or placing the meeting in a fictional virtual reality environment with avatars. Concerning evaluation, a pen-and-paper questionnaire can be digitized into a web questionnaire that calculates the results. A digital evaluation solution could enrich these data with psychophysiological measurements, mobile data, and electronic health records, or its execution could be responsive or gamified. The therapy-complementing materials, such as patient guides and CBT worksheets, can be shared via email or on the web. Meanwhile, their digital implementation could, for instance, make use of adaptive elements or take the form of a serious game to make them more engaging and effective. To recapitulate, we assert that the underlying function of the tools remains unchanged even when they grow more interactive, networked, and complex.

The results per function are described as a domain summary. They are as follows: (1) MHPs use complementary channels in client communication, (2) the evaluation of clients is being digitized, and (3) MHPs support therapeutic change using digital materials.

#### MHPs Use Complementary Channels in Client Communication

##### Face-to-Face Interaction

MHPs found many unique benefits with face-to-face interaction. Unmediated contact allowed for a superior connection with the client as the MHP could observe and react to nuances in client expressions and behavior that would otherwise be lost. This also enabled the MHP to generate a more reliable and accurate understanding of their problems, which was also valuable for psychological evaluation. Coming to the meeting in person also activated the client, which was found to be beneficial for clients with a tendency toward isolation and passivity. Finally, the in-person social interaction can be therapeutic in itself. An MHP facilitating group therapy for clients with social anxiety described the following:

The significant exposure is that you come to the group in person, and that you spend time with other people.

#15:62

The prevailing sentiment regarding the value of face-to-face meetings was succinctly described by an MHP working in psychiatry:

Of course, it [teletherapy] will never replace it [face-to-meetings]. We see a lot more than a person’s face when they arrive [to the practice]; there is the presence, the whole person.

#7:115

##### Telephone and SMS Text Messaging

MHPs used the telephone and SMS text messages to schedule meetings and checkups on their clients, and the telephone was also occasionally used for counseling. Only 2 MHPs reflected on the therapeutic potential of asynchronous messaging. One of them provided low-threshold support to their clients via WhatsApp, finding that merely exchanging messages could help them through a challenging situation and alleviate anxiety. An MHP with an occupational focus found that the time-independent nature of messages enhanced in-person therapy and allowed the therapy to “live in the mind” of their client between sessions. However, most MHPs’ client interactions occurred in scheduled meetings.

##### Teletherapy

The COVID-19 pandemic led many MHPs to convert some in-person meetings to a remote format, a practice that prevailed even after the pandemic. “The remote therapy has come to stay” (#15:39), as summarized by an MHP. Few MHPs explicitly preferred face-to-face meetings and were reluctant to schedule remote ones. The MHPs found remote meetings flexible and that they had the benefit of saving the client travel time. Remote meetings also facilitated a larger number of participants, both clients and MHPs, also from different locations. The decision between face-to-face therapy and teletherapy was often influenced by the client’s preference rather than readiness factors such as having a computer with a video camera and competence to use them. The readiness factors were only emphasized with some geriatric clients.

Interestingly, holding the meetings remotely did not necessarily change their content, indicating that they were digitized communication rather than natively digital. The videoconference meetings were found to be mediated counseling where, for instance, screen sharing had a similar function to a whiteboard in an in-person meeting. When the clients’ problems were not considerably debilitating, they could reflect on their behavior and be present in the relationship; remote counseling occurred very similarly to face-to-face meetings. An MHP conducting long-term psychotherapies reflected the following:

I can report that the therapy meeting works pretty much the same way. When I think back on the sessions, I don’t perceive a difference whether the session was conducted remotely or in person because the very same things happen, and it works in the same way.

#17:38

To summarize, we found that MHPs used media—face-to-face meetings, phone calls, messaging, and teletherapy—complementarily to serve different needs in the therapeutic relationship. The client preference and readiness influenced the medium chosen. However, it appeared that the different tools typically digitized the established practices rather than changing their content substantially.

#### The Evaluation of Clients Is Being Digitized

##### Overview

MHP responsibilities often included evaluation and counseling (Table 2). Psychiatric care routinely began with an evaluation period and continued with counseling. However, the balance between the 2 varied—some MHPs offered mainly counseling services, and one concentrated solely on psychological evaluation. Others blended evaluation and counseling in their work.

##### Pen-and-Paper Materials

MHPs routinely used pen-and-paper symptom questionnaires such as the Beck Depression Inventory [80] and Clinical Outcomes in Routine Evaluation-Outcome Measure [81] to evaluate the clients’ symptoms and track treatment progress. When a more extensive evaluation was needed, particularly on the client’s cognition, psychological tests were conducted, and in the sample, they were performed exclusively on pen and paper. Some organizations had “digitized” the questionnaires impromptu because of the coronavirus pandemic—they were sent to the clients via SMS text message or email, a practice that one MHP considered questionable.

##### Digital Platforms

A more sustainable solution for managing questionnaire data came from digital platforms that facilitate the collection, management, and storage of client data. Many organizations were picking up new systems to facilitate their work; however, the progress in their implementation varied alongside the MHP experiences of them. Some found the platforms beneficial and useful, whereas others were not equally impressed by their unwieldy implementation or were concerned that their older clients could not use them without support.

The study found that the digitalization of questionnaires and client data is underway in many organizations. Compared with the adoption of communication software, which was found to be necessary for the work, the adoption of questionnaire software appeared slower. In addition, the analog pen-and-paper materials that the MHPs were accustomed to using did not propose considerable drivers for change.

#### MHPs Support Therapeutic Change With Digital Materials

##### Overview

We found that most MHPs used some type of material to augment the effectiveness of counseling. The materials were used to give the client information—psychoeducation—on their disorder or condition, such as depression, anxiety, psychosis, sleeping, or pain. This aimed to develop the client’s confidence and capability to self-manage the symptoms. “We have to help the client to help themselves” (#14:127), explained an MHP on their philosophy regarding the materials. The visual materials also complemented the discussion-oriented therapeutic contact. They gave the client “something else than just talk” (#11:154): concrete materials and tools to use.

MHPs found that their clients’ reactions to the materials varied considerably. An MHP summarized the following:

Some think they are completely rubbish, useless slips of paper. Others think they are lovely: they appreciate that there is something concrete.

#11:153

An explanation for this difference was the variance in the clients’ interest in reading and their capability for self-reflection. To facilitate the adoption of the materials, the MHPs often presented them in the session, encouraged the client to explore them after the meeting at their own pace, and followed up on them.

##### Analog Materials

Some MHPs handed out paper brochures on disorders, and others handpicked materials from the internet or their own resources. The rationale for printing out or photocopying the materials was to make them more tangible and understandable, help the clients who do not have competency in finding the materials on the web themselves, and encourage clients to read the materials.

##### Digitized Materials

Almost all MHPs recommended digitized materials to their clients at least occasionally. The most common resource was the national MentalHub, which includes materials per disorder with separate content for youth. Its modular structure was found to be convenient, and the interactive sections and questionnaires were appreciated. In addition, third-sector services and materials, videos, and handpicked materials were recommended.

##### Mobile Apps

The MHPs rarely used or recommended mobile apps to their clients. When apps were used, the MHPs were personally familiar with them or the app was published by a credible public organization and targeted to the MHP clientele, as was the case with the youth-targeted Chillaa app.

##### DMHI Materials

Many MHPs had an indirect experience with DMHIs, most commonly with the national DMHI prescribed to their clients. They found that it could facilitate access to therapy for clients in sparsely populated areas, complemented the MHP know-how in specific domains, and was an option for new clients as they waited for therapist contact for “2-3 months” (#13:125) or “4 months” (#6:105).

Despite the advantages, the MHPs were generally at least somewhat hesitant and cautious regarding DMHIs. The nontransparent nature of the contents of the national DMHI discouraged it from being recommended. The MHPs perceived that DMHIs were most suitable for clients with relatively mild psychiatric problems, such as subclinical anxiety, stress, or relationship challenges. In contrast, many MHPs worked in special health care or with clients who had considerable clinical challenges and sought help because they could not manage their behavior without support. Thus, the clients needed and expected face-to-face reflection—“the ears and voice of the other person” (#6:119). The MHPs found that interpersonal contact—the therapeutic alliance between the MHP and the client—was constitutional, vital, healing, and remedial, and the lack of human interaction was the primary concern of almost all MHPs regarding DMHIs. If DMHIs were used, MHPs explicitly preferred supported over unsupported interventions. An MHP conducting long-term psychotherapy described the following:

Personal contact is of utmost importance. I believe that everyone may not need it, but the majority do. Some may get, at least for some time, help and relief from their socialization with a machine but it cannot replace a human.

#2:145

MHPs actively used materials in their client interactions. Across the media, the function of the materials was to help the client gain insights into their symptoms and ways to manage them. Therefore, we propose that the therapy-supporting materials could be viewed in a continuum from analog brochures to digitized self-help materials to (therapist-supported) structured DMHIs.

### What Characterizes the MHPs’ Digital Tool Use in Client Practice in General? (RQ 2)

#### Overview

**Figure 1:**
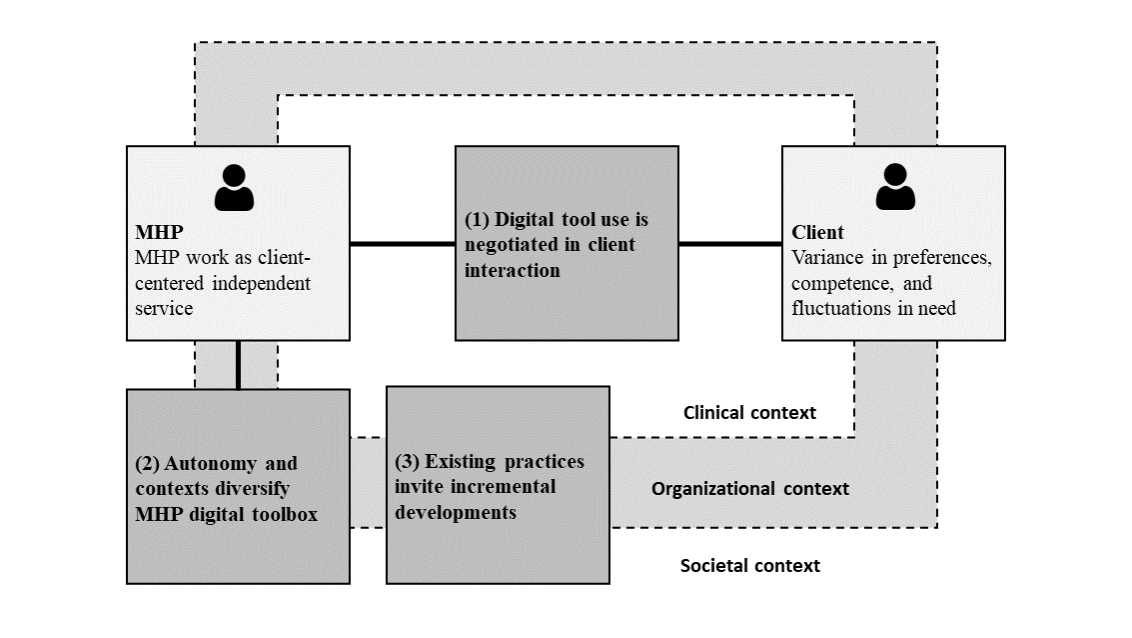
In total, 3 themes describe how mental health professionals (MHPs) used digital tools in client practice.

After analyzing how MHPs used particular digital tools, we examined their digital tool use in general in the context of their client work. The analysis of the interview data established three themes: (1) digital tool use is negotiated in client interaction, (2) autonomy and contexts diversify MHPs’ digital toolbox, and (3) existing practices invite incremental developments (Figure 1).

The three themes correspond with the nature of MHPs’ work, which we characterize as (1) client-centered, (2) independent, and (3) a service. MHPs exhibited client-centricity by being closely mindful of their clients and adjusting their digital tool use according to the perceived needs of their clients. This negotiation was shaped by the possibilities in the MHP toolbox. As MHPs had independence and autonomy in compiling their toolboxes, there was considerable heterogeneity in their contents and breadth. Third, the MHP work was a service—it focused on intangible interaction, was difficult to standardize, was produced and consumed simultaneously, and could not be stored [82]. The MHPs perceived that the digital tools augmented the interpersonal service they offered and, therefore, offered incremental rather than radical developments to the existing practices.

We describe how the MHP work was influenced by 3 layers of context. The clinical context included practices and expectations for flexible interpersonal MHP-client interaction. The organizational context may provide the MHP with tools such as digital platforms and processes for using them. The broader technological and cultural developments influenced the digital tools available in society and the MHP and client willingness and competency in using them.

#### Digital Tool Use Is Negotiated in Client Interaction

Almost all MHPs highlighted how they adjusted their behavior to their clients’ individual situations, needs, and symptoms in both evaluation and counseling. They adjusted, for instance, the focus of the evaluation; its duration; the frequency of counseling; and the therapeutic techniques, exercises, questionnaires, and digital tools used. MHPs may, for instance, offer their clients the possibility of choosing between face-to-face and remote meetings and whether a particular therapy was conducted in pen and paper or digitally assisted. Rather than following a rigid care routine, the MHPs found it vital to tailor the interaction to the client in the moment.

MHPs found negotiation and flexibility necessary because of the considerable variance in their clientele. Although they may work with a particular group of people, there was still substantial variation in the life context, symptoms, and needs of their clients; moreover, the clients’ situations may fluctuate. Thus, the key question in counseling was “finding the right tool at the right time” (#17:95). This position also reflected the nature of psychological problems—the MHP cannot directly influence the behavior of the client, who is ultimately responsible for the change. Flexibility also meant fostering client autonomy. An MHP described the following:

I have the overall approach that I offer the client different means, tools, and then they decide.

#1:83

Supporting client autonomy was also exhibited in how using a digital tool can be client-initiated and how MHPs considered client readiness and preferences in their tool recommendations. Overall, digital tools were perceived to serve higher-order therapeutic aims; they were “a means to an end” (#5:202).

Our research showed how MHPs prioritized establishing a working therapeutic relationship with their clients by adapting their behavior and the tools used. This reflected a client-centered profession, position, and practice.

#### Autonomy and Contexts Diversify the MHP Digital Toolbox

The MHP can only suggest exercises, materials, and tools that they know of, can access, and perceive as beneficial. The digital possibilities at the MHP disposal are referred to in this paper as the MHP digital toolbox, in which we found considerable variance. On one end, the digital toolbox was considerably limited—the MHPs used digitized and digital tools only to communicate with the client. “The only time when electricity flows through the wires is when we use the telephone” (#14:195), expressed an MHP of their nondigital care pathways. On the other end, MHPs used a breadth of communication channels, digitized and digital materials, and even apps and were aware of the national DMHI.

MHPs have the autonomy to compile their toolboxes. “When I am travelling, I may pick up something that I find works well for rehabilitation purposes: it can be a booklet, a game, or whatever” (#18:201), reflects an MHP. Thus, the MHPs’ attitude toward, interest in, and experience with digital solutions influenced the breadth of their digital toolbox. Those with more experience with digital tools showed higher competence and more positive attitudes toward them. Some MHPs proactively reflected on how they were generally curious about new digital tools, sought training on them, explored them on their own, and even participated in their development. Others exhibited a far more restricted and cautious stance on digital tools and self-perceived their digital skills as low and avoided their use.

Although MHPs have the autonomy to shape their digital toolbox, their freedom is limited and influenced by the possibilities in their organizational and societal context. Larger organizations often provided the MHPs with communication and questionnaire platforms and restricted them to the chosen platform, whereas MHPs working in their own practice had more liberty to choose these tools. The availability of credibly perceived digital tools, such as MentalHub psychoeducational materials, national DMHIs, third-sector resources, and some apps, encouraged their uptake. The external societal context also influenced the MHPs through their clientele—some clients requested remote meetings and introduced apps in counseling. Many MHPs found that their clients had the competency and means to use telehealth channels and they could search for and access digital content with little guidance, which was related to the digital tools being broadly used in Finnish society.

Digital tool use was influenced by the breadth of the MHP digital toolbox that the MHP can compile independently. Its contents were limited by the possibilities in the MHP organizational and societal context, and MHP attitudes, preferences, and experiences influenced tool uptake.

#### Existing Practices Invite Incremental Developments

Aside from the national DMHI, we found that the presently used and emerging tools brought incremental developments to the client practice. The teletherapy solutions reduced travel times but did not change the nature of counseling itself, the digitization of the pen-and-paper questionnaires allowed the same instruments to be filled on the web, and digitized psychoeducational materials served the same purpose as analog handouts. In other words, the digitized tools allowed the MHP to perform the tasks they already performed in an analog manner but more effectively. These developments retained the nature of MHP work as a service; did not offer the scalability benefits expected from digital interventions; and, therefore, did not directly address the insufficient resources in mental health care, which many MHPs were conscious of.

The MHPs’ principal hesitancy regarding digital treatments was their perceived insufficiency for their clients. Most MHPs highlighted how their clients had severe challenges and needed therapist interaction and that unsupported digital treatments were best suited for those with mild challenges. In addition, their clients sought counseling and expected interpersonal contact rather than a digital solution. Some MHPs were aware of how their position in the mental health ecosystem may have affected their thinking and attitudes. “This may be associated with my position in the treatment and service chain. It brings the view that [DMHI] was not enough and that what is needed is something longer and more intensive” (#8:144), an MHP pondered. In general, MHPs viewed that their professional service could not be replaced by a digital tool as its core lay specifically in human interaction.

Despite limitations, many MHPs could imagine the benefits that digital tools can offer in the future. They could extend the reach and access to therapy services in remote areas and offer specialized therapy services. They could lower the threshold to seek help and be helpful to clients who withdraw from others, are anxious and uncommunicative in MHP interaction, or have difficulties reflecting on their emotions verbally. Digital content could be more attractive; engaging; experience-rich; interactive; flexible; and, therefore, more effective than analog materials. Through their presence in the client’s everyday life, digital interventions may provide flexible support whenever and wherever the client wants and needs it; they could activate the client through notifications and adaptive exercises and give them encouraging, timely feedback. Overall, the digital possibilities could expand the treatment portfolio:

We need a large toolbox if we want to help everyone because people are so different. Also, they have different needs, different skills, and different capabilities to participate in something. So, we need a toolbox with a wrench, a screwdriver, all sorts of things.

#16:221

In summary, the digital tools implemented in existing client interaction–emphasizing practices were likely to contribute to incremental developments in MHP work. Although many MHPs were interested in new digital tools, their perspective was limited by their clientele, client expectations, and the nature of their work as a service.

## Discussion

### Principal Findings

It has been suggested that digital tools may aid in closing the mental health treatment gap. Unfortunately, even if effective, many new interventions fail in their implementation in complex real-life environments [83]. To facilitate this change, it is necessary to understand how MHPs use digital tools in clinical practice. Our study showed that digitized and digital tools were becoming a part of the clinical practice—MHPs used various channels in client interaction, the evaluation and diagnosis of clients were being digitized, and web-based materials were frequently used to complement counseling. The MHPs used the tools flexibly, adapting to their clients; there was variance in the breadth of MHP digital toolboxes; and the tools offered primarily incremental developments in MHP practice. These findings have vital implications for developing and implementing digital tools in mental health care.

### Contributions to Existing Research

Our research exhibited how MHPs use multiple channels to communicate with their clients. Most interviewed MHPs engaged their clients face-to-face and through telecounseling, a change that the COVID-19 pandemic has expedited [20-22]. Previous research has found that teletherapy offers convenience and flexibility [20], which we also identified as a motivator. We enrich previous findings by highlighting how the platforms for communication may be flexibly chosen per client and that there was considerable variance in MHP practices. Regarding messaging, for instance, only 2 MHPs explained how they used asynchronous messaging to support their clients. This finding invites consideration of how MHP services could be systematically augmented with, for instance, messaging [84] to improve treatment adherence and monitoring and offer support.

Previous research has found that MHPs perceive DMHIs as complementary to face-to-face therapies, prefer blended over stand-alone digital interventions, and consider unguided interventions insufficient for clients with substantial challenges [8]. We had similar findings and suggest that they may be explained by MHPs viewing the core of their work as lying in client interaction, where technologies hold a secondary, supportive role. This position reflects the history and nature of the profession and is supported by studies that have found the therapeutic alliance to be an essential common factor for therapeutic outcomes [12,85,86]. This also suggests that the digital transformation in mental health care may be driven by client and systemic needs rather than by MHP-driven motivators. The former includes the growing demand for mental health care, improving access to treatment in underserved areas, and offering an alternative to interpersonal treatments.

MHP attitudes toward digital tools may be explained by their experiences with them. Indeed, the 2 go hand in hand—attitudes are related to tool use [87,88], and use is related to more positive attitudes toward digital tools [9]. The direction of causality, however, remains unclear: do MHPs who consider the tools more positively use them more often, or do those who begin to use them grow more positive in their attitudes? Regardless, our research complements the findings by describing how positive attitudes and experiences might manifest in a broader digital toolbox whose contents can be used flexibly in client interaction. As the digital transformation progresses, this may lead to a growing divide between MHPs with extensive digital tools at their disposal and those who double down on the face-to-face approach.

The relationship between MHP attitudes and digital tool use may be conceptualized using the theory of planned behavior [89]. It posits that positive intentions are associated with the likelihood of the associated behavior occurring. Positive intentions, in turn, are influenced by the favorability of attitudes, positive social norms, and perceived ease or difficulty in performing the behavior. The theory has found empirical support in studies of MHP attitudes [87,90,91]—professionals are more likely to use digital tools when they view them favorably, their peers use them, and they find the tools easy to use, and these 3 factors can offer conceptual avenues for facilitating tool implementation.

### Implications for Digital Tool Development

The dominant mental health service delivery model reflects its psychotherapeutic roots—highly trained professionals offer services in one-on-one in-person settings [92]. However, as the need for mental health services grows, there is ever more awareness of the limitations of the delivery model—it lacks scalability as it is closely tied to a scarce human resource, MHP time. However, uncoupling time from the service delivery challenges the very fundament the services are based on, the interpersonal therapeutic alliance [11]. Our research suggests that MHPs may view telehealth solutions and digitized questionnaire suites more favorably than digital interventions as the former complements personal interaction, whereas the latter challenges it. We surmise that these attitudes may extend to other technological developments such as artificial intelligence, chatbots, social media, and virtual reality [7,93], which may be viewed as disrupting the beneficial qualities of the therapeutic relationship unnecessarily.

**Figure 2:**
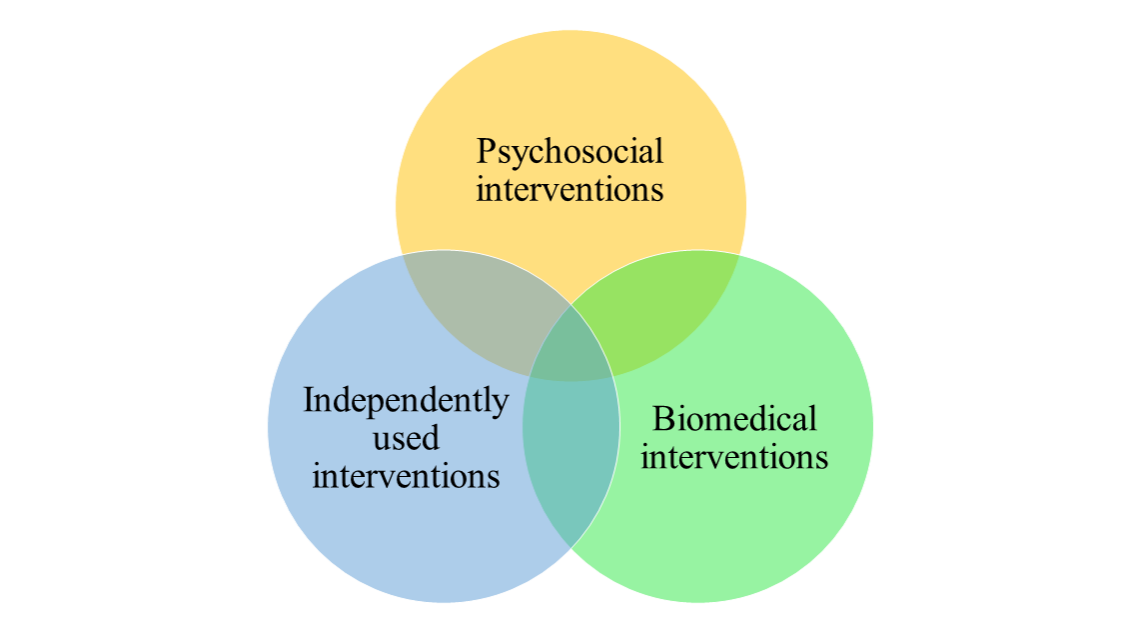
Differentiating between 3 modes of treatment. Psychosocial interventions are based on interpersonal interaction, biomedical interventions affect the nervous system, and independently used interventions encourage change through learning.

We suggest consciously distinguishing 3 modes of treatment (Figure 2) to alleviate the tension between them. Psychosocial interventions such as counseling are founded on interpersonal interaction and the therapeutic relationship [11,85,86]; biomedical treatments such as psychiatric medication and electroconvulsive therapy affect the nervous system; and independently used interventions such as psychoeducational courses [23] and digital interventions [94] are based on clients acquiring new skills, building motivation, and creating opportunities to change [95]. Psychosocial interventions are flexible, interpersonal, and adaptive to the client, but on a societal scale, their benefits are tied to the available MHP resources. Psychiatric medications are scalable but may have side effects. Independently used interventions can offer best practice psychoeducation and guidance at a scale whenever and wherever, yet they lack the empathetic and motivating interpersonal connection.

Differentiating between the different modes of treatment can help clients and clinicians perceive their complementary potential. Associating an independently used intervention with a web-based program or course instead of therapy may help establish realistic expectations and facilitate reaching clients who expect and benefit from such an approach. Interestingly, we found that many MHPs already encouraged their clients to access analog or digitized materials between sessions. Thus, some MHPs appear to have adopted a blended approach [36] where the treatment uses both psychosocial and publicly available, independently used components. However, the self-adopted practices lacked consistency, which invites the consideration of systemic ways to improve service processes.

Focusing on psychosocial interventions, our model (Table 3) allows for the differentiation of which function—communication, evaluation and diagnosis, and facilitating therapeutic change—the digital tool relates to. This allows developers and health care management to advance a user-centered position and connect the solution with existing practices and MHP and client needs. However, further research is needed to understand how to harmonize the 3 modes of treatment in clinical practice so that they complement each other throughout the client journey.

Training and education are commonly recommended to improve MHP digital tool adoption and use [10,39,96-99]. This individual-focused approach is synergistic with the autonomy and independence that MHPs enjoy in their work, which may also diminish the impact of group- or organizational-level change efforts [100]. We maintain that training may be sufficient to implement digitized tools that suggest incremental changes in clinical practices. In contrast, the digital tools that present radical changes to the modus operandi must be accompanied by substantial structural changes. Several frameworks may be helpful in this regard [101]. They include the Consolidated Framework for Implementation Research [8,83]; Promoting Action on Research Implementation in Health Services [102,103]; and the Nonadoption, Abandonment, Scale-up, Spread, and Sustainability framework [104,105]. All these frameworks highlight how creating a usable digital tool is not enough—it needs to be considered in terms of the adopters, clinical practices, organizational care processes, and societal context. Our work complements these models by illuminating the MHP-client implementation context (Figure 1).

### Limitations

This research was conducted in Finland, and the results showed that the external context influences MHP behavior and attitudes. Finland is highly developed digitally [106]. Of the adult population, 93% use the internet [107], and web-based interaction with governmental services is very common [106]. In health care, e-services, including e-prescriptions, are commonly used, and public and many private services have integrated their patient data into a shared repository called Kanta [108]. The status of DMHIs for depression has been legitimized by their acceptance into the national clinical practice guidelines [109]. Aligned with the theory of planned behavior [89], the societal digital development and broad use of digital devices in health care are likely to influence MHP and client attitudes positively and contribute to their adoption.

The study recruitment efforts sought to attract participants from various backgrounds as well as those who held a more critical stance on digital tools. However, the sample included only a few critical voices and several MHPs with substantial knowledge of digital tools and therapies. The research theme likely attracted those with a more positive stance on the topic and may not represent the entire MHP population. The interviewees included psychologists, psychotherapists, nurses, and an occupational therapist who worked in various contexts, with clients of different ages, and with clients who had various disorders. One MHP was experienced in conducting a national DMHI. The sample did not include physicians or MHPs from private occupational health care or basic-level health care for nonstudents, providing avenues for future research efforts. In addition, the study does not necessarily reflect the positions of the health care leadership who may not directly work with clients and are responsible for managing and developing the service and information systems used within them. Further research may be needed to understand the leadership position and strategy and consider the change in management efforts required to implement the solutions in organizational settings [109].

### Conclusions

New digital tools are being actively developed in mental health care, and scalable solutions are expected to alleviate the global mental health problem. This study illuminated the context of MHPs, who play a crucial role in adopting and implementing new technologies in client interaction. Our research showed that MHP work involves 3 key functions: communicating with the client, diagnosing and evaluating them, and facilitating therapeutic change. Teletherapy was widely accepted and adopted alongside other media, and the evaluation of clients was becoming more digitized. Digitized psychoeducational materials were widely used, but MHPs hesitated to recommend stand-alone digital therapies that were perceived as insufficient for their clients.

We characterized the MHP work as a client-centered independent service. The MHPs adjusted the techniques, interactions, and tools used per client. The MHPs had the independence and autonomy to choose the tools used from a range of possibilities. This created heterogeneity in the digital toolboxes, which were influenced by MHP preference, organizational context, and tools available in the external environment. The digital tools introduced to this service context often proposed incremental rather than radical developments, considerably limiting their impact. More research is needed to examine when and how scalable, independently used interventions can best complement psychosocial interventions and for whom they may work independently.

## Appendix

Multimedia Appendix 1 The interview guide.

## Abbreviations

CBT: cognitive behavioral therapy
COREQ: Consolidated Criteria for Reporting Qualitative Research
DMHI: digital mental health intervention
HUS: Helsinki University Hospital
MHP: mental health professional
RQ: research question
