# Supplementary material for: Factors Affecting Digital Tool Use in Client Interaction According to Mental Health Professionals: Interview Study
Source: JMIR Hum Factors. 2023 Jul 10;10:e44681. doi: 10.2196/44681 (PMC10366964; doi:10.2196/44681)
Supplement: Multimedia Appendix 1 [file humanfactors_v10i1e44681_app1.docx]

# Appendix 1

## Interview overview

- **Introduction.** The interviewer tells the interviewee about the study and the interview process.
- **Background.** Establishing rapport with the interviewee. Familiarizing with the interviewee context of work and clientele.
- **Digital tools.** Exploring the interviewee usage and attitudes regarding digital tools in their client practice.
- **Digital games.** Exploring the interviewee attitudes regarding digital games and how they are discussed in client interaction.
- **Closing.** Establishing shared understanding, snowballing, and closing the interview.

The five phases are described in more detail below with possible follow-up questions. The interviewer is encouraged to flexibly follow-up on the relevant themes the subject brings up during the interview while maintaining the general structure of the interview.

## Interview structure

- **Introduction**
  - **Interviewer introduction.** The interviewer introduces themselves by name, title, and relationship with the research.
  - **Interview purpose.** The purpose of the interview is explained to the interviewee: the aim is to understand how MHPs use digital tools in their client-work to facilitate their research and development, and to understand how MHPs perceive digital games.
  - **Informed consent.** The interviewee is told that the information regarding the study, as also given in the survey, apply to the interview. For instance, the interviewee can stop the interview at any time without giving a reason. Any interviewee questions regarding the study are answered. Informed consent is confirmed.
  - **Recording.** The interviewee is asked permission for recording the interview.
  - **Structure.** The subject is explained the structure of the interview.
    - The interviewee’s work context and clientele is explored to give context to their answers.
    - The interviewee usage and experiences regarding digital tools is explored.
    - It is explored how digital gaming is reflected in client interaction
- **Interviewee background.**
  - **Education.** What is the interviewee education?
  - **Work context.**Where does the MHP work?
  - **Clientele.** Who are their clients?
- **Digital tools.**
  - **Usage.** Does the interviewee use any digital tools, materials, or technologies with their patients?
  - **Experiences.** What kind of experiences have they had with them?
  - **Most value.** What creates value in the tools to them and their clients?
  - **Least value.** What doesn’t?
  - **Expectations.** What would they expect from digital tools?
- **Digital games**
  - **Attitude.** What does the interviewee generally think about digital games?
  - **Usage.** Do they or their close ones play digital games? What do they think about it?
  - **Gaming in clients.** Do the interviewee’s clients play? How is gaming reflected in the clinical practice?
- **Closing**
  - **Reflective summary.** The interviewer gives a reflective summary of the key findings to the interviewee which are reflected together to confirm shared understanding of the interview.
  - **Questions.** The interviewer answers any questions from the interviewee.
  - **Snowballing.** The interviewer asks if the interviewee would like to share information about their study to their professional acquaintances.
